# A Dual-Task Paradigm Using the Oral Trail Making Test While Walking to Study Cognitive-Motor Interactions in Older Adults

**DOI:** 10.3389/fnagi.2021.712463

**Published:** 2021-09-13

**Authors:** Antoine Langeard, Marta Maria Torre, Jean-Jacques Temprado

**Affiliations:** ^1^Aix-Marseille Université, CNRS, ISM, Institut des Sciences du Mouvement, Marseille, France; ^2^Normandie Univ, UNICAEN, INSERM, COMETE, Caen, France

**Keywords:** dual-task, switching, gait, younger old, older old

## Abstract

**Objective:** With aging, gait becomes more dependent on executive functions, especially on switching abilities. Therefore, cognitive-motor dual-task (DT) paradigms should study the interferences between gait and switching tasks. This study aimed to test a DT paradigm based on a validated cognitive switching task to determine whether it could distinguish older-old adults (OO) from younger-old adults (YO).

**Methods:** Sixty-five healthy older participants divided into 29 younger-old (<70 years) and 36 older-old (≥70 years) age groups were evaluated in three single-task (ST) conditions as follows: a cognitive task including a processing speed component [Oral Trail Making Test part A (OTMT-A)], a cognitive task including a switching component [Oral Trail Making Test part B (OTMT-B)], and a gait evaluation at normal speed. They were also evaluated under two DT conditions, i.e., one associating gait with OTMT-A and the other associating gait with OTMT-B. Cognitive and gait performances were measured. The comparison of cognitive and gait performances between condition, logistic regression, and receiver operating characteristic (ROC) analyses were performed.

**Results:** The cognitive and gait performances were differently affected by the different conditions (i.e., ST, DT, OTMT-A, and OTMT-B). The OTMT-B produced higher interference on gait and cognitive performances. Moreover, a higher number of errors on the OTMT-B performed while walking was associated with the older-old age group.

**Conclusion:** Using validated cognitive flexibility tasks, this DT paradigm confirms the high interference between switching tasks and gait in older age. It is easily implemented, and its sensitivity to age may highlight its possible usefulness to detect cognitive or motor declines.

## Introduction

With aging, the cognitive and motor function impairments coexist and are often the early markers on the pathway to neurodegeneration, loss of autonomy in the activities of daily living, decrease in mobility, and risk of falls. For a long time, age-related declines in cognitive and motor performance were assessed separately. The underlying assumptions were that, on the one hand, cognitive abilities are powerful indicators of the declines in motor capacities and the risks of falls in older age (Langeard et al., 2019a, 2020) and, on the other hand, mobility impairments (e.g., slowing of gait speed) are more likely to increase the risk of fall when they are associated with the alterations of cognitive performance (Montero-Odasso et al., 2012). These findings are consistent with the hypothesis that cognitive and motor functions become more and more interrelated in older adults so that the alterations of motor behavior (e.g., slowing of movement execution) share common causes with cognitive declines by virtue of the dedifferentiation process (Baltes and Lindenberger, 1997; Sleimen-Malkoun et al., 2013, 2014). This has been demonstrated for information processing speed in target-aiming tasks (Sleimen-Malkoun et al., 2013), for inhibition processes in bimanual coordination (Temprado et al., 2020), and switching capacities [i.e., flexibility between tasks or mental sets (Miyake and Friedman, 2012] during walking (Langeard et al., 2019b).

Accordingly, changes in motor-cognitive interaction are widely used as a marker to assess age-related changes in functional behaviors, such as postural control and gait. While walking at a comfortable pace is an automatic motor task that hardly requires cognitive functions in physically and cognitively robust adults, the relationship between cognitive function and gait performance is strengthened in dual-task (DT) situations (Al-Yahya et al., 2011). Moreover, it has been shown that the sensorimotor control of gait patterns becomes more dependent on cognition during aging, leading to increased competition between cognitive and motor processing when performed simultaneously (Schäfer et al., 2006). This is of high interest since, in older adults, DT performance, particularly when walking simultaneously to perform specific cognitive tasks, is associated with a higher risk of falls, fall recurrence, and pathological cognitive decline (Beauchet et al., 2008; Montero-Odasso et al., 2017). Accordingly, identifying a valid DT paradigm that could be incorporated into clinical and research settings is an important issue.

The classical tests, such as the “Stop Walking when Talking Test” (Lundin-Olsson et al., 1997), developed from the clinical experience that some frail elderly patients stop walking when they start a conversation, are often used to detect older adults at risk of fall. However, it has finally shown inconsistent results in predicting fall risks, possibly due to the lack of standardized instructions regarding the attention-demanding task (i.e., “engaging in a conversation;” Beauchet et al., 2009). Moreover, a recent meta-analysis reported no significant association between cognitive-motor interference (deterioration in cognitive, motor performances, or both, when performed simultaneously) and falls due to the large variety of cognitive tasks used in DT paradigms (Wollesen et al., 2019). Various cognitive tasks are currently used. They require either visuomotor abilities, memory recall, working memory, or executive function (Riby et al., 2004; Patel et al., 2014). Of interest, tasks relying on executive processes produced greater DT interferences (Riby et al., 2004; Patel et al., 2014). However, a well-developed rationale is still lacking to determine what are the most appropriate cognitive tasks to be selected. Consequently, the tasks were not chosen often based on their possible interactions with gait. The high dependence of gait on cognitive flexibility (Langeard et al., 2019b) does lend credence to the interest of including these executive processes in DT paradigms through validated cognitive tasks.

A suitable choice, in this respect, is the Trail Making Test (TMT), which is known for relying on switching abilities and has demonstrated its validity to assess them during aging (Sánchez-Cubillo et al., 2009). Interestingly, the TMT is predictive of effectiveness in the instrumental activities of daily living (Cahn-Weiner et al., 2002) and gait capacities (Hirota et al., 2010) in community-dwelling older adults. These findings support the possible interrelation between gait and TMT performance, which can be evaluated through the analysis of the interferences produced when simultaneously performing these two tasks.

Alexander et al. (2005) were the first to propose a “Walking Trail Making Test (WTMT)” to test stepping accuracy under increased conditions of concurrent cognitive and visual demands (Alexander et al., 2005; Perrochon et al., 2014; Schott, 2015; Klotzbier and Schott, 2017; Wei et al., 2019). In these studies, participants were instructed to follow a fixed pathway as fast and accurately as possible, stepping on targets (TMT-1), stepping on targets with an increasing sequential number (TMT-2, i.e., 1-2-3…), or those with increasing sequential number and letters (TMT-3, i.e., 1-A, 2-B, 3-C…). They detected a strong association between spatial navigation performance and cognitive dysfunction in older adults (Alexander et al., 2005; Perrochon et al., 2014). While these studies confirmed the relationship between mental flexibility and mobility during aging, they did not evaluate cognitive-motor interferences as a DT paradigm would. A limit of this paradigm is that the TMT used includes additional visual and motor components, which may also interfere with the walking task. Actually, to our knowledge, no previous study has evaluated the cognitive-motor interferences produced by a DT paradigm in which the cognitive part was a validated mental flexibility task, for instance, the TMT.

A possible solution lies in the association between Oral TMT (OTMT) and walking. The OTMT, which was initially developed by Ricker et al. (1996), not only allows removing the visual and motor components of the WTMT task but also, in addition, is a valid, discriminative, and practical measurement tool in healthy older adults (Bastug et al., 2013). To the best of our knowledge, OTMT has been scarce, if ever, associated with a walking task to investigate motor-cognitive interference during gait in older adults. This study addressed this issue in the elderly either younger than 70 years old [younger-old adults (YO)] or 70 years old and older [older-old adults (OO)].

Baltes and Smith mentioned that most people maintain their level of everyday intelligence (relevant to the problems people face daily) until around the age of 70 years (Baltes and Smith, 2003). Moreover, spontaneous walking speed is relatively stable up to the age of 70 years. The critical age for significant deleterious change in walking speed has been estimated to be around 70 (Ferrucci et al., 2016). It has also been suggested that DT performance may differ between older adults of different age groups. The cognitive-motor interference during DT is more pronounced in subjects older than 70 years compared with other age groups (Magnani et al., 2021). Pothier et al. (2015) reported no difference between YO and young adults in a Multiple Object Tracking While Walking DT, under low attentional load conditions. In contrast, their performance was altered and became similar to OO during high attentional load conditions (Pothier et al., 2015). It has been suggested that differences between OO and YO could be mediated by the decline in switching abilities (Langeard et al., 2019b). Accordingly, a DT paradigm loading these abilities, for instance, the Walking Oral Trail Making Test (W-OTMT), is expected to be more heuristic to distinguish OO from YO than classic DT paradigms. This experiment aimed to test this hypothesis in healthy older adults. Specifically, due to the W-OTMT, we expected to distinguish participants of 70 years and more from participants below 70 years through longer response time to the cognitive task, more errors, and, at motor level, stronger perturbations of gait patterns.

## Methods

### Participants

Participants were recruited from advertisements in local newspapers. The inclusion criteria were as follows: (1) to be able to walk 10 m without aid and (2) to be able to read, understand, and provide informed consent. Participants with uncorrected vision or auditory impairments and known neurological, psychiatric, or vestibular conditions were excluded from the study. Age, level of education, global cognition [through the Montreal Cognitive Assessment, known to be more sensitive to subtitle cognitive deficits than the Mini-Mental State Examination (Trzepacz et al., 2015)], and Hand Grip strength [as a proxy measure of global physical performance (Stevens et al., 2012)] were collected. Participants were then divided into two groups, i.e., OO when they were 70 years old and above and YO when they were below 70 years old (Baltes and Smith, 2003). These characteristics of the participants are presented in Table 1. This study was conducted in accordance with the Declaration of Helsinki, and the protocol has been approved by the French National Ethics Committee (CPP IDF10 no. 2019-A03263-54).

**Table 1:** The characteristics, cognitive performances, and differences between age groups [presented as mean (SD)] of the participants.

	**Younger-old (29)**	**Older-old (36)**	**Total (65)**	**Younger-old vs**. **older-old**	
				***t***	***p***
Age (years)	67.00 (1.51)	73.56 (3.66)	70.63 (4.37)	−9.033	<0.001
Sex (% of women)	56.2%	44.4%	49.2%	−0.852	0.398
Years of education (years)	11.65 (2.99)	12.42 (2.30)	12.08 (2.64)	−1.161	0.250
Hand Grip (kg)	36.79 (10.74)	37.06 (11.06)	36.94 (10.84)	−0.098	0.922
MoCA (30)	26.03 (2.30)	25.64 (3.31)	25.82 (3.08)	0.512	0.610

*MoCA, Montreal Cognitive Assessment*.

### Measurements

Gait was evaluated in single-task (ST) and DT conditions following the published guidelines for spatiotemporal gait analysis in older adults (Kressig and Beauchet, 2006). The evaluation of gait was performed through the GaitRite® system (Clifton, NJ, USA), a system validated for determining spatiotemporal gait parameters. Participants wore comfortable footwear. The data collection was performed in a quiet, closed room with no auditory or visual interference. Participants started walking 2 m before reaching the walkway and stopped 2 m beyond it. The instructions were standardized, and participants were asked to walk at their usual speed: “Please, could you walk at a normal pace, as you would do on the street. Are you ready? Go.” Ten trials were performed at their usual gait speed. Gait velocity (cm.s^−1^), step length (cm), and step time (s) were recorded and averaged from the 10 trials. Step length variability was calculated based on the SD of the step length divided by the average step length, representing a spatial variability index (Lovie, 2005). These measures are often used in gait analysis research, are sensitive to age, risk of falls, and cognitive decline, and have been affected during dual-tasking (Nakamura et al., 1996; Pothier et al., 2015; Ko et al., 2018; Bessot et al., 2020). The gait measurements were averaged across the two legs, and no separate analysis was carried out for each leg since we had no hypothesis about gait symmetry.

**Figure 1:**
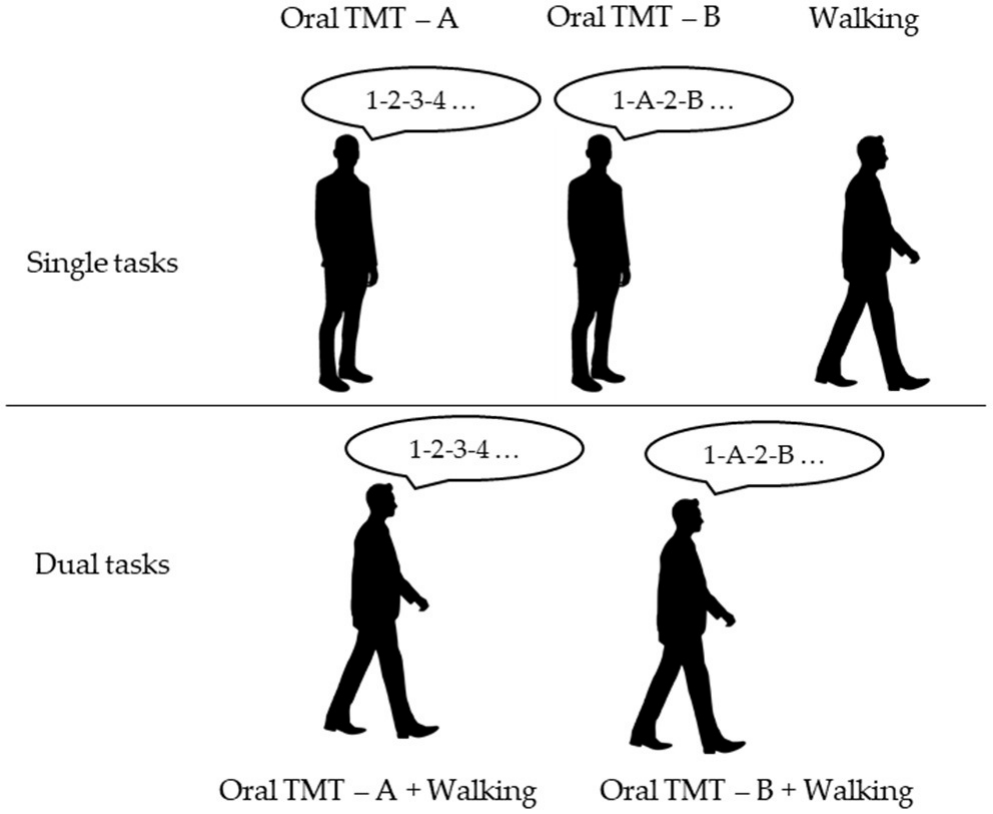
Experimental design of the novel paradigm (TMT-A, Trail Making Test part A; TMT-B, Trail Making Test part B).

The cognitive task performed during walking was a commonly used and validated cognitive task (Kressig and Beauchet, 2006), i.e., the OTMT (Mrazik et al., 2010). We used a similar DT procedure as those administered by Ho et al. (2019), though it was more complete. In fact, in this study, both cognitive scores (i.e., number of errors and time of completion) in ST and DT and gait parameters (i.e., gait velocity, step length, and step time) were recorded (Ho et al., 2019). Five conditions were proposed to the participants in a randomized order. The instructions were standardized. Three ST conditions were administered as follows: (1) walking at spontaneous speed, (2) OTMT part A while standing (OTMT-A) (i.e., the following instruction was given to the participant: “Please, could you count from 1 to 25, loudly, as quickly and accurately as possible: 1, 2, 3, etc. Are you ready? Go”), and (3) OTMT part B while standing (OTMT-B) (i.e., the following instruction was given to the participant: “Please, could you count from 1 to 25, loudly, as quickly and accurately as possible from a number to a letter, specifically, 1-A-2-B-3-C, and so on, Are you ready? Go”). In part A, participants were asked to count as fast as possible with the lowest possible number of errors from 1 to 25. In part B, participants were asked to alternate numbers and letters, starting with 1-A-2-B-3-C…, until the administrator asked to stop, i.e., when the participants completed 25 answers. The DT trials were composed of two conditions as follows: (1) the DT-OTMT-A, in which the participants were asked to perform the OTMT-A task while walking and (2) the DT-OTMT-B, in which the participants were asked to perform the OTMT-B task while walking (Figure 1). One trial was performed for each of the four conditions involving cognitive tasks. The test was stopped when the participants gave 25 answers.

For the OTMT-A, the W-OTMT-A, the OTMT-B, and the W-OTMT-B, the time of completion corresponding to the time of the participants to give 25 answers was recorded. The 25 answers were collected, and the number of errors was calculated. An error occurred when the participants gave an incorrect answer (e.g., six instead of five, or D instead of C). A shift in the sequence of answers after an error was not considered an error (e.g., 1-A-2-C-3-D… only counted as one error). Participants were informed that they would not be stopped if they made an error and were asked to continue walking even if they made an error. No instruction was provided concerning the task to prioritize (i.e., gait or cognition; Kelly et al., 2013). Gait and cognitive performance are presented in Tables 2, 3, respectively. The repartition of the cognitive scores in ST and DT among participants is presented in Supplementary Figure 1.

**Table 2:** Gait parameters under single-task (ST) and dual-task (DT) conditions.

		**YO**	**OO**	**Total**
Single-Task	Velocity (cm/s)	125.32 (15.97)	128.72 (17.84)	127.24 (17.00)
	Step length (cm)	67.71 (6.75)	68.19 (7.09)	67.98 (6.89)
	Step time (s)	0.544 (0.042)	0.535 (0.046)	0.539 (0.045)
	Step length variability	0.030 (0.006)	0.031 (0.007)	0.031 (0.007)
Dual-Task (OTMT-A)	Velocity (cm/s)	122.89 (24.77)	122.15 (25.70)	122.47 (25.11)
	Step length (cm)	64.66 (8.47)	64.71 (8.46)	64.69 (8.39)
	Step time (s)	0.537 (0.061)	0.544 (0.086)	0.0541 (0.075)
	Step length variability	0.054 (0.048)	0.056 (0.027)	0.055 (0.037)
Dual-Task (OTMT-B)	Velocity (cm/s)	87.02 (24.67)	86.75 (25.69)	86.87 (25.05)
	Step length (cm)	58.32 (8.49)	58.42 (7.69)	58.38 (7.98)
	Step time (s)	0.710 (0.159)	0.747 (0.276)	0.730 (0.231)
	Step length variability	0.076 (0.057)	0.078 (0.056)	0.078 (0.056)
Dual-Task cost of switching (DT-OTMT-B/DT OTMT-A)	Velocity (cm/s)	0.71 (0.13)	0.70 (0.14)	0.71 (0.13)
	Step length (cm)	0.90 (0.07)	0.90 (0.06)	0.90 (0.06)
	Step time (s)	1.31 (0.24)	1.36 (0.39)	1.34 (0.33)
	Step length variability	1.98 (1.76)	1.74 (1.49)	1.84 (1.60)

*YO, younger-old adults; OO, older-old adults; OTMT-A, Oral Trail Making Test part A; OTMT-B, Oral Trail Making Test part B*.

**Table 3:** Times and scores of the Oral Trail Making Test (OTMT).

			**YO**	**OO**	**Total**
OTMT-A	Time (s)	ST	7.42 (2.34)	7.27 (1.83)	7.33 (2.05)
		DT	8.70 (2.15)	9.12 (2.54)	8.94 (2.36)
	Errors (*n*)	ST	0.00 (0.00)	0.00 (0.00)	0.00 (0.00)
		DT	0.04 (0.20)	0.09 (0.51)	0.07 (0.41)
OTMT-B	Time (s)	ST	24.01 (8.26)	26.29 (9.16)	25.28 (8.77)
		DT	24.65 (9.07)	26.59 (7.23)	25.73 (8.09)
	Errors (*n*)	ST	1.15 (1.77)	1.03 (1.66)	1.08 (1.70)
		DT	1.11 (1.40)	2.56 (1.93)	1.92 (1.85)^*^
Switching cost (OTMT-B/OTMT-A)	Time (s)	ST	3.38 (1.18)	3.72 (1.31)	3.57 (1.25)
		DT	2.92 (1.07)	3.08 (1.03)	3.01 (1.04)
	Accuracy (/25)	ST	0.95 (0.07)	0.96 (0.07)	0.96 (0.07)
		DT	0.96 (0.06)	0.90 (0.07)	0.93 (0.07)^*^
DT-Cost of switching (DT switching cost/ST switching cost)	Time (s)		0.95 (0.42)	0.911 (0.44)	0.93 (0.43)
	Accuracy (/25)		1.01 (0.11)	0.94 (0.08)	0.97 (0.10)^*^

*YO, younger-old adults; OO, older-old adults; ST, single-task; DT, dual-task; OTMT-A, Oral Trail Making Test part A; OTMT-B, Oral Trail Making Test part B*.

* *Significant predictor of age group*.

A switching cost on completion time and accuracy was calculated in ST and DT by dividing the OTMT-B scores by the OTMT-A scores in these two conditions. This score is a strong indicator of task-switching abilities (Sánchez-Cubillo et al., 2009). Moreover, a DT cost of switching on completion time and accuracy was calculated by dividing the switching cost in the DT condition by the switching cost in the ST condition. This score captures the impact of performing a switching task while walking on cognitive capacities. Finally, a DT cost of switching was also calculated for gait variables (i.e., velocity, step length, step time, and step length variability) by dividing the gait measures obtained in W-OTMT-B by the measures obtained in W-OTMT-A. This score captures the impact of performing a switching task while walking on gait performance.

### Statistics

#### Characteristics of the Participants

The characteristics of the participants were compared according to their age group (YO vs. OO) using the independent *t*-tests, after the verification of homogeneity of variances using Levene's test.

#### DT Interferences

To assess the interferences produced when performing the W-OTMT on gait spatiotemporal parameters and cognitive scores in the whole sample, the repeated measure ANOVAs were used to compare cognitive scores (i.e., completion time and the number of errors) between condition (ST vs. DT) and TMT parts (part A vs. part B) and to compare gait performance (i.e., velocity, step length, step time, and step length variability) between conditions (ST vs. DT-TMT-A vs. DT-TMT-B). The Bonferroni corrections were applied to *post-hoc* multiple comparisons. Cohen's *d* was provided as the measurement of effect sizes with *d* = 0.2 considered a “small effect size,” 0.5 a “medium effect size,” and above 0.8 a “large effect size” (Cohen, 1992).

#### The Distinction Between YO and OO

To determine whether the DT paradigm distinguished OO from YO, logistic regressions were used. They allowed determining whether age groups could be distinguished based on the cognitive and gait performances. The receiver operating characteristic (ROC) curve analyses were performed in the presence of a significant model to determine the model accuracy and possible cutoffs in gait and cognitive scores. The analyses were performed using IBM SPSS Statistics 25 (IBM Corp., Released in 2017, IBM SPSS Statistics for Windows, version 25.0. Armonk, NY, USA), and the statistical significance was set at *p* = 0.05.

## Results

### Characteristics of the Participants

No differences in sex, education, MoCA, and Hand Grip strength were found between YO and OO (Table 1). Therefore, no covariates were used in the following models.

### DT Interferences

The repeated measure ANOVAs tested the interferences produced on gait spatiotemporal parameters and cognitive scores of the whole sample when performing the W-OTMT.

### DT Interferences-Cognition Analysis

**Figure 2:**
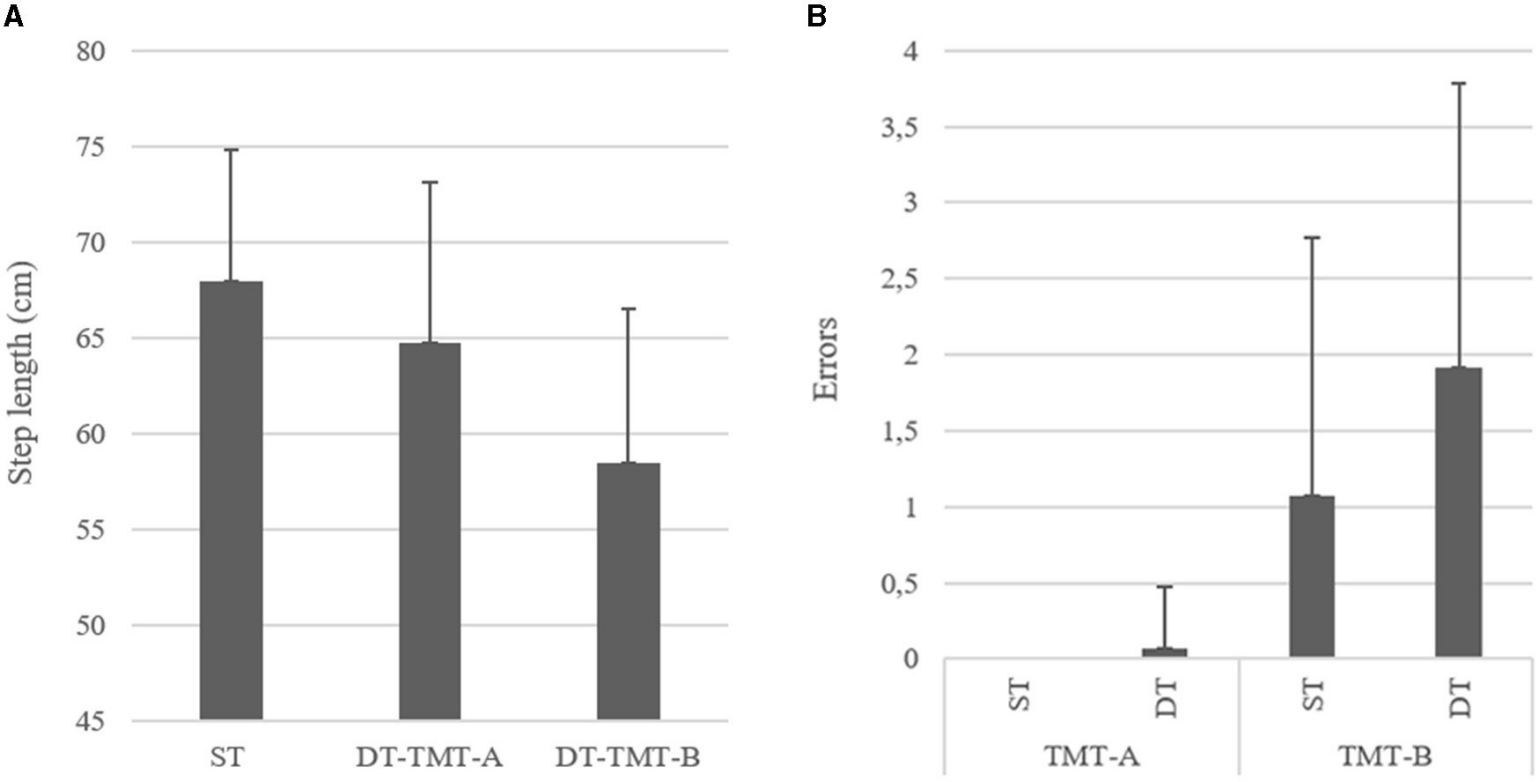
**(A)** Left and **(B)** right, respectively, showing the differences between conditions on errors on the cognitive tests and step length (cm) in the whole sample (TMT-A, Trail Making Test part A; TMT-B, Trail Making Test part B; ST, Single Task; DT, Dual-Task).

The repeated measure ANOVAs found an effect of condition (ST vs. DT) on the TMT times. DT times were higher than ST of 1.011 s (*F* = 5.89, *p* = 0.018, *d* = −0.314). Similarly, errors were more numerous (of 0.458 errors) in DT condition compared with ST (*F* = 8.683, *p* = 0.005, *d* = 0.380). An effect of the TMT part (A vs. B) was also found on times, with a longer time of 17.305 s during part B compared with part A (*F* = 319.764, *p* < 0.001, *d* = −2.309). Similarly, the number of errors was higher (of 1.458) in part B compared with part A (*F* = 67.43, *p* < 0.001, *d* = 1.060). The *post-hoc* analysis further detected that, in the TMT-A, the number of errors in ST and DT was not statistically different (*p* = 1.00), while in the TMT-B, participants made significantly more errors in DT condition (+0.850 errors, *t* = 4.015, *p* < 0.001) (Figure 2A).

### DT Interferences-Gait Analysis

Gait velocity, step time, step length, and step length variability were also affected by the condition (ST vs. W-OTMT-A vs. W-OTMT-B) (*F* = 142.826, *p* < 0.001; *F* = 86.287, *p* < 0.001; *F* = 50.102, *p* < 0.001; and *F* = 23.841, *p* < 0.001, respectively). Gait velocity was slower in W-OTMT-B compared with W-OTMT-A and ST (−35.305 cm/s, *t* = −13.378, *d* = 1.723, *p* < 0.001 and −39.843 cm/s, *t* = 15.437, *d* = 1.945, *p* < 0.001, respectively). Step length differed between the three conditions, W-OTMT-A step length was smaller than ST (−3.217 cm, *t* = 4.384, *d* = 0.552, *p* < 0.001), and W-OTMT-B step length was smaller than W-OTMT-A (−6.262 cm, *t* = 8.533, *d* = 1.075, *p* < 0.001) (Figure 2B). Step length variability differed between the three conditions, W-OTMT-A step length variability was higher than ST (0.025 cm, *t* = 3.608, *d* = 0.455, *p* < 0.001), and W-OTMT-B step length variability was smaller than W-OTMT-A (0.023 cm, *t* = 3.295, *d* = 0.415, *p* < 0.001). Finally, step time was longer for W-OTMT-B compared with ST and W-OTMT-A (+0.189 s, *t* = 8.704, *d* = −1.097, *p* ≤ 0.001 and +0.188 s, *t* = 8.634, *d* = 1.088, *p* ≤ 0.001, respectively).

### The Distinction Between YO and OO

**Figure 3:**
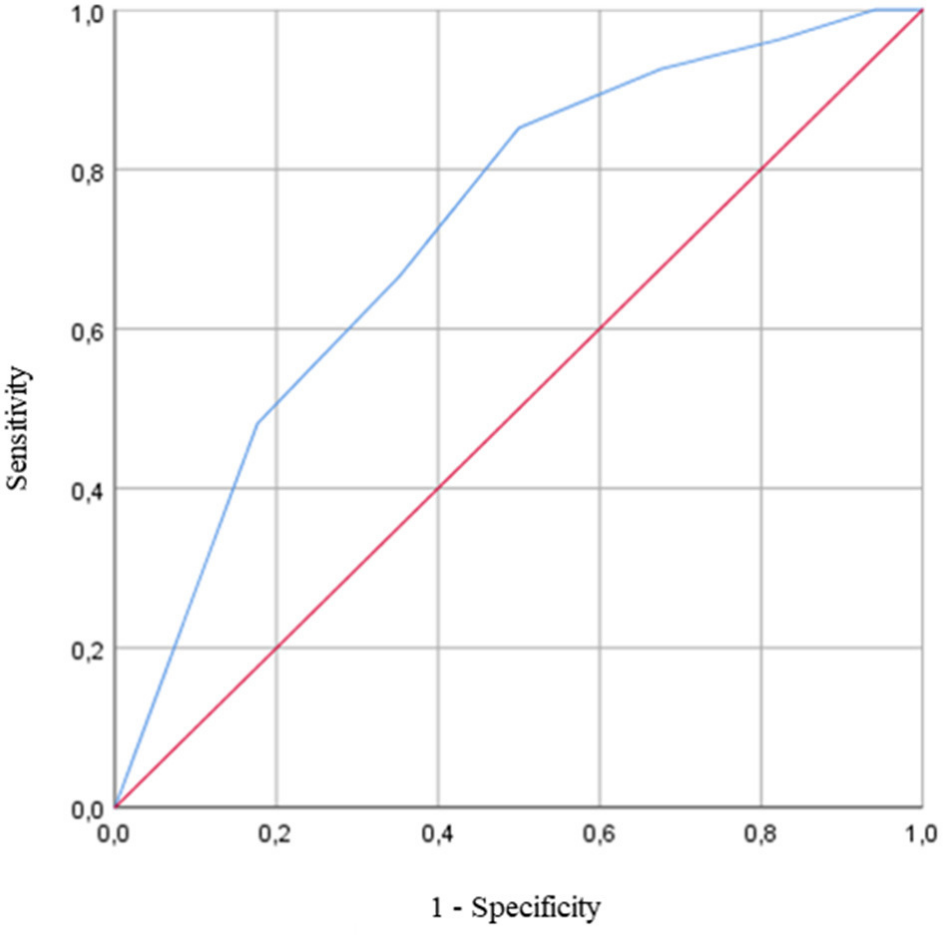
Receiver operating characteristic (ROC) curve for the prediction of the age group based on the number of errors on the Oral Trail Making Test part B (OTMT-B) while walking [area under the curve (AUC) = 0.725 ± 0.065, *p* = 0.003]. The AUC is equal to 1 for perfect discrimination and 0.5 for an uninformative cutoff point. The optimal cutoff is defined as the value that combined the best combination of sensitivity and specificity.

Logistic regressions were then used to determine if the DT paradigm could distinguish OO from YO. The logistic regression analysis found no significant association between all the ST cognitive and gait performances and age groups. In DT conditions, the number of errors on the OTMT-B significantly predicted the age group (χ^2^ = 10.366, *p* < 0.001), and participants with more errors were significantly more likely to be OO participants [odds ratio (OR): 1.683 (1.183, 2.396), *p* = 0.004]. The ROC curve analysis confirmed the model accuracy [area under the curve (AUC) = 0.725 ± 0.065, *p* = 0.003, sensitivity = 0.647, specificity = 0.667] and detected those participants who committed two errors and above were more likely to belong to the OO group (Figure 3).

Consistently, the switching cost on accuracy in DT condition also predicted the age group (χ^2^ = 10.308, *p* < 0.001), and participants with higher costs on accuracy in DT conditions were significantly more likely to be OO participants [OR: −13.379 (−22.612, −4.14), *p* = 0.004]. The ROC curve analysis confirmed the model accuracy (AUC = 0.734 ± 0.065, *p* < 0.001, sensitivity = 0.647, specificity = 0.692) and detected those participants who reduced the accuracy of 8.55% and above when going from DT-OTMT-A to DT-OTMT-B were more likely to belong to the OO group.

Finally, the DT cost of switching on accuracy could also distinguish OO from YO (χ^2^ = 7.502, *p* = 0.006). The ROC curve analysis confirmed the model accuracy (AUC = 0.695 ± 0.068, *p* < 0.001, sensitivity = 0.882, specificity = 0.346) and detected those participants who increased their switching cost on the accuracy of 8.16% and above when going from ST to DT were more likely to belong to the OO group. The characteristics of the models are presented in Tables 4–6.

**Table 4:** Characteristics of the logistic regression model of cognitive and gait performances under ST conditions.

		**BIC**	**χ^2^**	***p***
H0		87.870		
OTMT-A	Time (s)	93.541	0.082	0.774
	Errors (*n*)	No variance		
OTMT-B	Time (s)	90.924	1.057	0.304
	Errors (*n*)	91.906	0.075	0.785
Switching cost (OTMT-B/OTMT-A)	Time (s)	90.783	1.198	0.274
	Errors (*n*)			
Gait	Velocity (cm/s)	95.400	0.638	0.424
	Step length (cm)	91.640	0.080	0.424
	Step time (s)	95.359	0.679	0.410
	Step length variability	94.901	1.137	0.286

*H0, null hypothesis; BIC, bayesian information criterion*.

**Table 5:** Characteristics of the logistic regression model of cognitive and gait performances under DT conditions.

		**BIC**	**χ^2^**	***p***
H0		87.870		
DT-OTMT-A	Time (s)	89.838	0.459	0.498
	Errors (*n*)	90.055	0.242	0.623
DT-OTMT-B	Time (s)	91.085	0.896	0.344
	Errors (*n*)	81.614	10.366	0.001
DT-Switching cost (DT-OTMT-B/ DT-OTMT-A)	Time (s) Errors (*n*)	89.784 75.898	0.324 10.308	0.569 0.001
DT-OTMT-A	Velocity (cm/s)	94.319	0.014	0.907
	Step length (cm)	94.332	0.001	0.982
	Step time (s)	94.186	0.147	0.702
	Step length variability	94.291	0.042	0.838
DT-OTMT-B	Velocity (cm/s)	96.036	0.002	0.966
	Step length (cm)	96.036	0.002	0.960
	Step time (s)	95.626	0.412	0.521
	Step length variability	96.017	0.021	0.885

*H0, null hypothesis; BIC, bayesian information criterion*.

**Table 6:** Characteristics of the logistic regression model of DT costs on cognitive and gait performances.

		**BIC**	**χ^2^**	***p***
H0		87.870		
DT cost of switching on cognition (Switching cost in DT/Switching cost in ST)	Time (s) Errors (*n*)	90.181 82.795	0.116	0.734 0.006
DT cost of switching on gait (DT-OTMT-B gait/DT-OTMT-A gait)	Velocity (cm/s) Step length (cm) Step time (s) Step length variability	94.288 94.333 94.021 93.981	0.045 0.000 0.312 0.352	0.832 1.00 0.577 0.553

*H0, null hypothesis; BIC, bayesian information criterion*.

No other variable could distinguish OO from YO.

## Discussion

This study aimed to test a cognitive-motor DT paradigm, based on a validated switching cognitive task (i.e., the OTMT), to (1) analyze the interferences it produces on the cognitive and gait abilities of older adults and (2) determine whether it could distinguish OO from YO.

The DT interferences were observed in both the cognitive (TMT) and motor (gait) domains, particularly when walking, and the OTMT-B (i.e., a task strongly relying on switching abilities) was performed simultaneously. Specifically, more errors were observed on the cognitive task, while smaller steps were produced in this DT condition, compared with the ST or the DT associating walking and the OTMT-A. Thus, this study allowed (for the first time to our knowledge) isolating the effects of cognitive flexibility from the other cognitive processes and the motor components (biomechanical, sensorimotor, etc.) also involved in the DTs. W-OTMT-A incorporates a rhythmic component (related to gait and speech) and loads information processing speed component (related to the TMT-A itself). W-OTMT-B also incorporates these different components in addition to flexibility. Therefore, it can be hypothesized that the results observed for only W-OTMT-B, and the calculated costs, reflected specific processes, independent of the common processes currently implicated in both W-OTMT-A and W-OTMT-B. Accordingly, we contended that they are closer to a pure representation of the interference produced by the flexibility component of W-OTMT-B (Sánchez-Cubillo et al., 2009). By showing that interferences occurred between the switching component of a cognitive task and gait when performed simultaneously, these results confirmed previous findings suggesting that, among the executive subdomains, switching abilities are implicated in gait control in older adults (Langeard et al., 2019b).

Of interest, most of the DT performances (cognitive and motor) were affected by the W-OTMT-B, but participants reduced their step length even in the W-OTMT-A condition. It suggests that, in older adults, other cognitive functions involved in OTMT-A (including processing speed) could also play an essential role in gait control (Martin et al., 2013). These results are in contrast with those observed by Nadkarni et al. (2010), who used two types of cognitive DT, one relying on working memory processes (N-back) and the other loading spatial attention abilities. They reported that dual-tasking affected the performances of older adults regardless of the task, suggesting that changes in gait may be a function of limited attentional resources irrespective of the type of cognitive task (Nadkarni et al., 2010). Conversely, this result suggests the existence of task specificity in DT interferences.

Several theories have been formulated to explain declines in DT performance, including the bottleneck theory and the capacity-sharing theory. On the one hand, according to the bottleneck theory of DT, the tasks are involved in serial processing and must be processed sequentially, thereby resulting in lowering speed or performances in one or both tasks (Pashler, 1994). On the other hand, the capacity-sharing model assumes that the processing of multiple tasks can proceed simultaneously, but there is a limited capacity to perform two operations simultaneously, so that the processing capacities may be allocated to one task over the other (Tombu and Jolicœur, 2003). We contended that the bottleneck theory cannot explain that, in this study, the effect of dual-tasking on gait was task-specific (more important for the OTMT-B than the OTMT-A). Instead, our results are more in accordance with the capacity-sharing theory of dual-tasking. Moreover, the capacity-sharing theory is also consistent with the dedifferentiation hypothesis of motor-cognitive aging, according to which aging not only leads to the structural and functional alterations of individual components of the neuromusculoskeletal system but also results in the systemic reorganization of interactions between domains, in particular between cognitive and motor domains (Sleimen-Malkoun et al., 2014). The dedifferentiation hypothesis supports that cognitive and motor domains may share more common processes with aging (Sleimen-Malkoun et al., 2014) and, therefore, triggers age-related declines in DT performances.

This interpretation is also supported by the results observed in our DT paradigm, which allows distinguishing OO from YO. Specifically, the number of errors performed at OTMT-B while walking differentiated between OO and YO, and also between the DT costs and switching cost on errors. These results suggest that the number of errors at the OTMT-B was related to the switching component of the task. Thus, our DT paradigm appears sensitive to distinguish age groups and could also allow detecting premature cognitive aging in future studies. In this aim, it might suffice to count the number of errors when performing the W-OTMT-B and to detect the participants that show OO-like performance. In other words, the W-OTMTB performance could be used to predict an age group (70 years and above) known to be at risk of important cognitive (Baltes and Smith, 2003), gait (Ferrucci et al., 2016), and DT decline (Magnani et al., 2021) (rather than a continuous age) and, therefore, to detect premature aging. In addition, it has the advantage of being easily implemented for both clinical and research purposes. However, further research should determine whether the W-OTMT is more sensitive than other DT paradigms to detect the risk of falls or the onset of functional cognitive decline and, eventually, dementia (Verghese et al., 2002; Beauchet et al., 2008; Montero-Odasso et al., 2017). In fact, in this study, the lack of functional measures and fall-related outcomes could be considered a limitation of the statistical models. Nevertheless, thanks to the ROC curve analysis, we highlighted those two or more errors on the W-OTMT-B could characterize OO, so that the occurrence of such scores in younger populations could be a signal indicating the onset of cognitive or motor aging.

Moreover, the fact that a higher number of errors on the W-OTMT-B were associated with older age is also consistent with the age-related cognitive-motor differentiation hypothesis, which could occur for specific processes (Sleimen-Malkoun et al., 2013). Interdependencies between sensorimotor and cognitive processes become accentuated during aging. Thus, sharing limited cognitive and motor resources between two concurrent conditions should result in age-related decreased DT performances (Schaefer and Schumacher, 2010; Sleimen-Malkoun et al., 2014). Other DT studies converged to a similar conclusion arguing that, with aging, sensory and motor functions are increasingly taxing cognitive control (Lindenberger et al., 2000). This is also consistent with the Compensation-Related Utilization of Neural Circuits Hypothesis (CRUNCH), according to which more neural resources are needed at an older age to achieve equivalent performance to that of younger adults, leading to possible compensation when the cognitive demand is lower (single-tasking) but to age differences when the cognitive demand increases (dual-tasking; Reuter-Lorenz and Cappell, 2008; Fettrow et al., 2021).

Consistent with previous research, DT interference was only observed in the cognitive domain but not in the motor domain (i.e., gait variables). Previous studies that explored the differences between OO and YO under DT conditions by using different DT paradigms also reported similar gait performances between the two age groups under DT conditions (Camicioli et al., 1997; Lindenberger et al., 2000; Pothier et al., 2015). This was observed for visuospatial tracking DT, memorizing while walking DT, or walking while performing a verbal fluency DT (Camicioli et al., 1997; Lindenberger et al., 2000; Pothier et al., 2015). These results could reflect motor control strategies. It is well-known that when no instruction about task priority is given (as in this study), older adults often give priority to postural control (Li et al., 2001). However, the lack of effects of DT on gait variables could also result from the relatively low number of steps performed during the DTs, due to the short duration of the OTMT. It could be responsible for the low reliability of the gait parameters, in particular of the gait variability. Thus, further study would evaluate the W-OTMT during more trials, although learning effects could highly impact their results.

## Conclusion

The present W-OTMT paradigm allows investigating the subtle cognitive mechanisms involved during walking in older adults, which are critical from both clinical and research points of view. We also showed that W-OTMT is sensitive to small age differences in older adults. Altogether, our results demonstrated that this specific DT paradigm is promising for evaluating cognitive-motor interaction and possibly detecting premature cognitive and motor declines.

## Data Availability Statement

The raw data supporting the conclusions of this article will be made available by the authors, without undue reservation.

## Ethics Statement

The studies involving human participants were reviewed and approved by French National Ethics Committee (CPP IDF10). The patients/participants provided their written informed consent to participate in this study.

## Author Contributions

AL and J-JT: conceptualization. J-JT: validation, supervision, and funding acquisition. AL: formal analysis and writing the original draft preparation. AL, MT, and J-JT: investigation and writing the article. AL and MT: data curation. All the authors have read and agreed to the published version of the manuscript.

## Funding

This research was supported by the Active Aging 2.0 Research Chair from Institut des Sciences du Mouvement de la Faculté des Sciences du Sport de Marseille and AG2R-LA MONDIALE.

## Conflict of Interest

The authors declare that the research was conducted in the absence of any commercial or financial relationships that could be construed as a potential conflict of interest.

## Publisher's Note

All claims expressed in this article are solely those of the authors and do not necessarily represent those of their affiliated organizations, or those of the publisher, the editors and the reviewers. Any product that may be evaluated in this article, or claim that may be made by its manufacturer, is not guaranteed or endorsed by the publisher.

## Supplementary Material

Supplementary Figure 1 Representation of the repartition of the percentage of participants according to the number of correct answer to the single-task oral trail making test part-A (ST OTMT-A), to the dual-task oral trail making test part-A (DT OTMT-A), to the single-task oral trail making test part-B (ST OTMT-B), and to the dual-task oral trail making test part-B (DT OTMT-B).
