# Supplementary figures and images for: A Dual-Task Paradigm Using the Oral Trail Making Test While Walking to Study Cognitive-Motor Interactions in Older Adults
Source: Front Aging Neurosci. 2021 Sep 13;13:712463. doi: 10.3389/fnagi.2021.712463 (PMC8475182; doi:10.3389/fnagi.2021.712463)

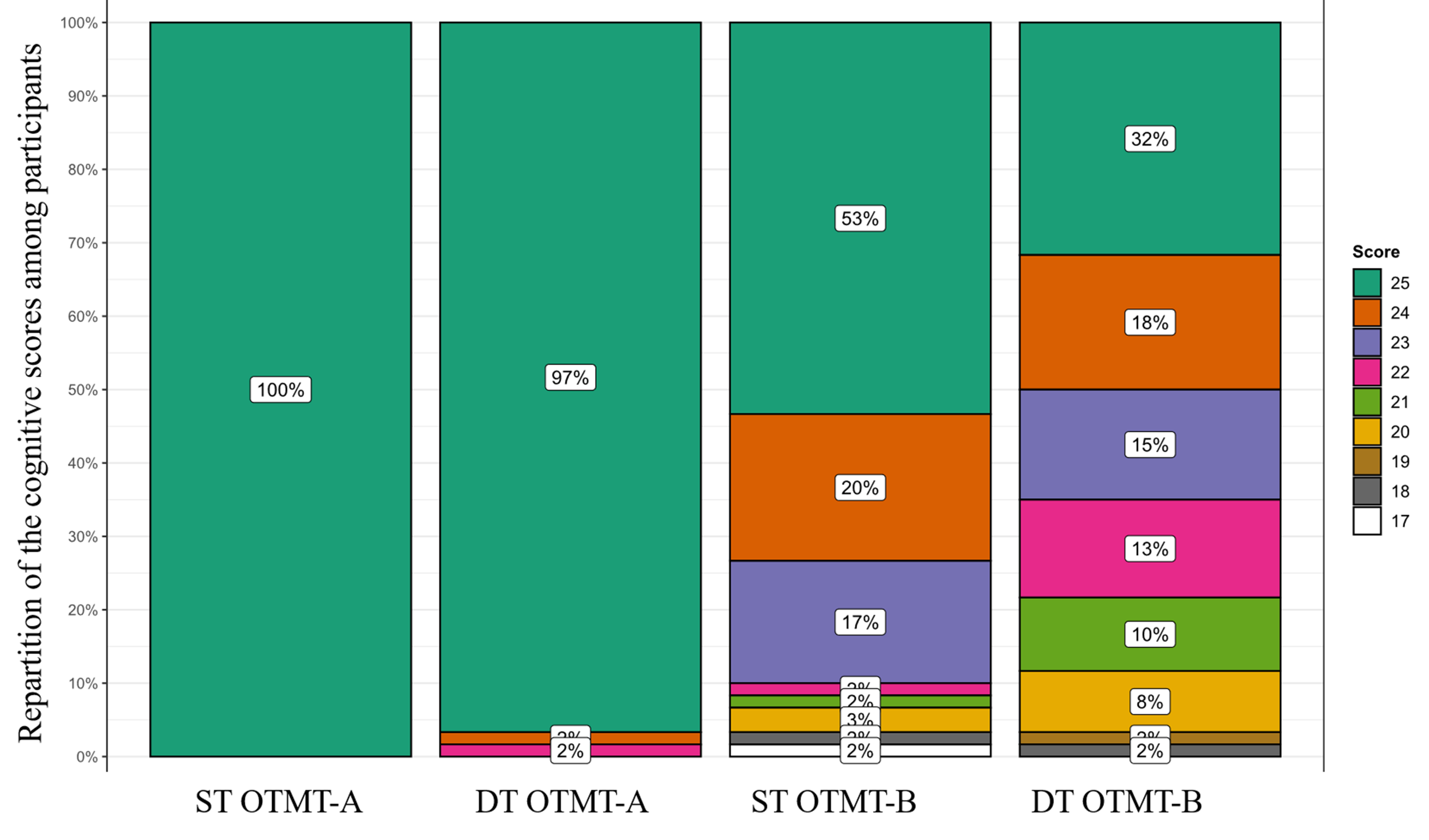

Supplement: Supplementary Figure 1 — Representation of the repartition of the percentage of participants according to the number of correct answer to the single-task oral trail making test part-A (ST OTMT-A), to the dual-task oral trail making test part-A (DT OTMT-A), to the single-task oral trail making test part-B (ST OTMT-B), and to the dual-task oral trail making test part-B (DT OTMT-B). [file Image_1.TIF]
